# Supplementary material for: Networked Cluster Formation via Trigonal Lipid Modules for Augmented Ex Vivo NK Cell Priming
Source: Int J Mol Sci. 2024 Jan 26;25(3):1556. doi: 10.3390/ijms25031556 (PMC10855780; doi:10.3390/ijms25031556)
Supplement: Supplementary file 1 [file ijms-25-01556-s001.zip › ijms-2815818-supplementary.pdf]

Supporting Information

Networked Cluster Formation via Trigonal Lipid Modules for Augmented Ex Vivo NK Cell Priming

Jaewon Park, Sungjun Kim, Ashok Kumar Jangid, Hee Won Park and Kyobum Kim *

Department of Chemical & Biochemical Engineering, Dongguk University, Seoul 22012, Republic of Korea; dbffldkssk5@gmail.com (J.P.); sungjun.kim@dgu.ac.kr (S.K.); ashok4483@gmail.com (A.K.J.); hana4339@gmail.com (H.W.P.)

* Correspondence: kyobum.kim@dongguk.edu

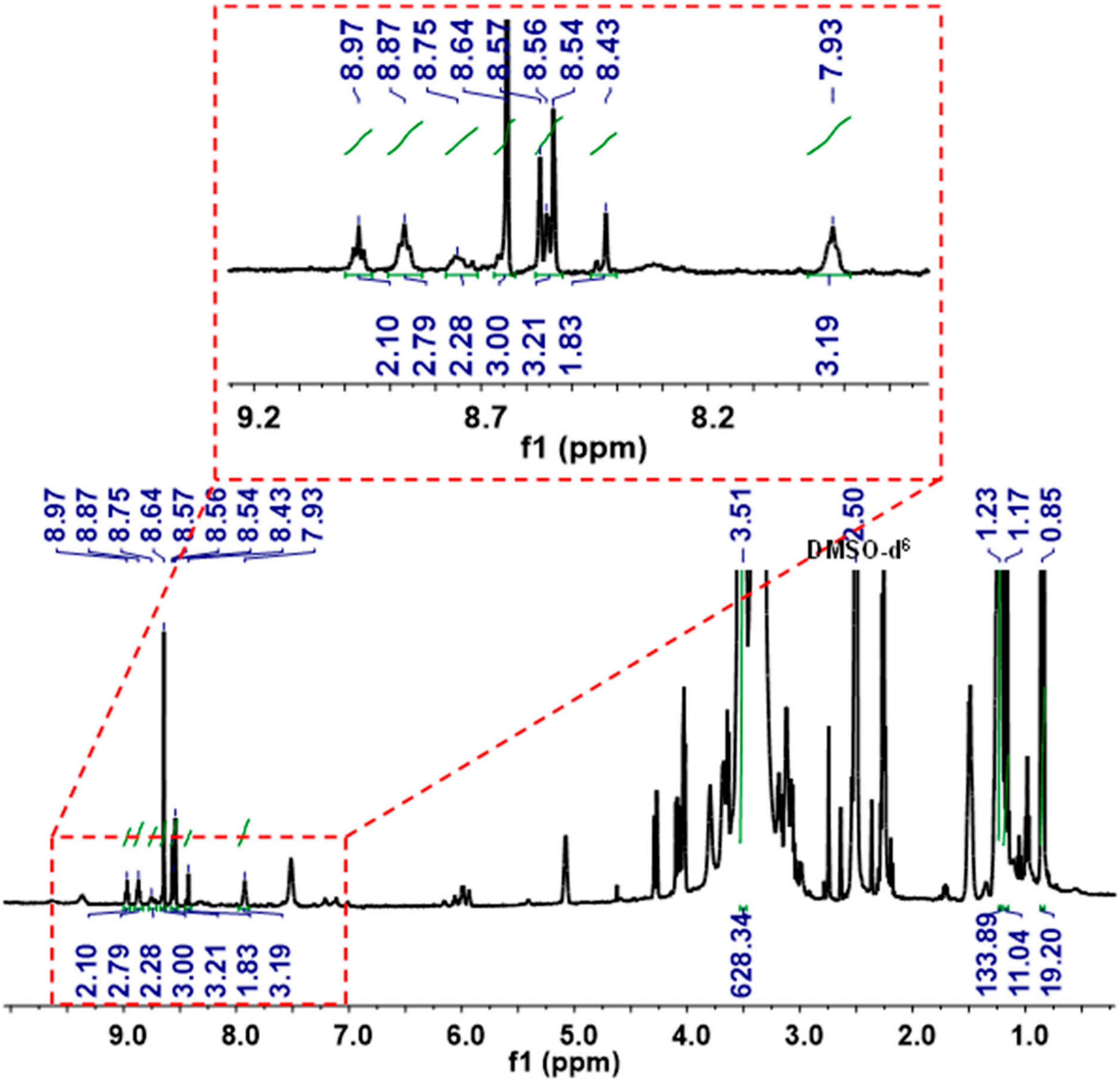

Figure S1. Proton nuclear magnetic resonance (^{1}H-NMR) spectra of DSPE-PEG-NH branching with TMA ((DSPE-PEG-NH)$_3$-T). The NMR spectra result was recorded in DMSO-d6 solvent.
